# Time dependency and unique etiology of barotrauma in COVID-19: A retrospective cohort study with landmark analysis and pathological approach

**DOI:** 10.1371/journal.pone.0282868

**Published:** 2023-03-15

**Authors:** Takafumi Kabuto, Ryutaro Seo, Chisato Miyakoshi, Yuri Shimizu, Yusuke Shima, Daisuke Yamashita, Shigeo Hara, Ryosuke Hirabayashi, Keisuke Tomii, Masakazu Takayama, Keisuke Tetsumoto, Masao Saito, Hiroshi Hamakawa, Bela Suki, Yutaka Takahashi

**Affiliations:** 1 Department of Thoracic Surgery, Kobe City Medical Center General Hospital, Kobe, Hyogo, Japan; 2 Department of Emergency Medicine, Kobe City Medical Center General Hospital, Kobe, Hyogo, Japan; 3 Department of Research Support, Center for Clinical Research and Innovation, Kobe City Medical Center General Hospital, Kobe, Hyogo, Japan; 4 Department of Pathology, Kobe City Medical Center General Hospital, Kobe, Hyogo, Japan; 5 Department of Respiratory Medicine, Kobe City Medical Center General Hospital, Kobe, Hyogo, Japan; 6 Department of Biomedical Engineering, Boston University, Boston, Massachusetts, United States of America; Sapienza University of Rome: Universita degli Studi di Roma La Sapienza, ITALY

## Abstract

**Background:**

Barotrauma frequently occurs in coronavirus disease 2019. Previous studies have reported barotrauma to be a mortality-risk factor; however, its time-dependent nature and pathophysiology are not elucidated. To investigate the time-dependent characteristics and the etiology of coronavirus disease 2019-related-barotrauma.

**Methods and findings:**

We retrospectively reviewed intubated patients with coronavirus disease 2019 from March 2020 to May 2021. We compared the 90-day survival between the barotrauma and non-barotrauma groups and performed landmark analyses on days 7, 14, 21, and 28. Barotrauma within seven days before the landmark was defined as the exposure. Additionally, we evaluated surgically treated cases of coronavirus disease 2019-related pneumothorax.

We included 192 patients. Barotrauma developed in 44 patients (22.9%). The barotrauma group’s 90-day survival rate was significantly worse (47.7% vs. 82.4%, p < 0.001). In the 7-day landmark analysis, there was no significant difference (75.0% vs. 75.7%, p = 0.79). Contrastingly, in the 14-, 21-, and 28-day landmark analyses, the barotrauma group’s survival rates were significantly worse (14-day: 41.7% vs. 69.1%, p = 0.044; 21-day: 16.7% vs. 62.5%, p = 0.014; 28-day: 20.0% vs. 66.7%, p = 0.018). Pathological examination revealed a subpleural hematoma and pulmonary cyst with heterogenous lung inflammation.

**Conclusions:**

Barotrauma was a poor prognostic factor for coronavirus disease 2019, especially in the late phase. Heterogenous inflammation may be a key finding in its mechanism. Barotrauma is a potentially important sign of lung destruction.

## Introduction

Barotrauma, pneumomediastinum/subcutaneous emphysema and pneumothorax in intubated patients are caused by alveolar rupture due to a pressure difference caused by mechanical ventilation [1,2]. This is a well-known poor prognostic factor among clinicians; however, the incidence rate is relatively low (0.5–13%) [1–4].

Coronavirus disease 2019 (COVID-19) has emerged as a worldwide pandemic, and many critically ill patients with COVID-19 receive invasive mechanical ventilation in the intensive care unit (ICU) [5,6]. Barotrauma often occurs in patients with COVID-19 during mechanical ventilation and is also an assumed mortality-risk factor [4,7–10]. Previous studies have shown that COVID-19-related pneumonia more frequently causes barotrauma in patients with invasive mechanical ventilation than other diseases [4]. However, barotrauma cannot be considered a risk factor at the time of intubation because it occurs during mechanical ventilation. Therefore, an understanding of its time-dependent effects is warranted. Moreover, the reason barotrauma occurs more frequently in patients with COVID-19 has not been elucidated, and few studies have assessed its mechanism and etiology [7,10].

Therefore, this study aimed to investigate the time-dependent impact of barotrauma on the prognosis of patients with COVID-19 and concomitant barotrauma compared with that of those without barotrauma. We also considered its pathophysiology using the clinical findings of COVID-19-related pneumothorax.

## Methods

### Study design and setting

This retrospective cohort study and the case series study used data from Kobe City Medical Center General Hospital and was approved by the Institutional Review Board of Kobe City Medical Center General Hospital (zh211209, Nov 26th, 2021). The need for written informed consent was waived by providing the patients with a means of opting out. Oral and written informed consent was obtained from all patients in the case series (Case 1: 28^th^ July 2021, Case 2: 21^st^ February 2022, Case 3: 16^th^ February 2022).

Our institution is a tertiary referral center in Kobe City, in Hyogo Prefecture, and has preferentially received critically ill patients with COVID-19 since March 2020. By the end of May 2021, 14,924 patients and 39,705 patients had been diagnosed with COVID-19 in Kobe City and Hyogo Prefecture, respectively [11,12]. In this period, COVID-19 vaccination had not yet reached ordinary people in Japan.

### Data collection

We retrospectively reviewed intubated patients aged > 20 years with COVID-19 in our institution from March 2020 to May 2021. We identified patients with COVID-19 from the electronic medical records. These patients were diagnosed with COVID-19 using nasal-swab reverse transcription polymerase chain reaction testing, the loop-mediated isothermal amplification method, or an antigen test. Patients who were not intubated were excluded and patients treated with extracorporeal membrane oxygenation (ECMO) were included because they were intubated prior to receiving ECMO therapy.

Barotrauma, pneumomediastinum/subcutaneous emphysema and pneumothorax, was diagnosed by evaluating the medical records or radiological images, such as those from chest radiography and computed tomography, during the mechanical ventilation period. The COVID-19 patients with the history of intubation were classified into the barotrauma group and the non-barotrauma group by the experience of barotrauma during the intubation period. Pneumomediastinum/subcutaneous emphysema and pneumothorax after extubation were not considered barotrauma in this study.

Clinical data, including patient profile data, comorbidities, examination results, mechanical ventilation variables, treatment contents, radiological images, intraoperative findings, and pathological information, were collected from our electronic medical records. We collected mechanical ventilation and arterial blood gas data in the morning after intubation and in the morning of barotrauma onset (only in the barotrauma group). We also collected data on anti-COVID-19 treatments, including corticosteroids, during hospitalization. Tension pneumothorax was defined as pneumothorax found due to respiratory failure, not due to chance or routine imaging follow-up. The intubation period was defined as the length from intubation to successful extubation, or ventilator withdrawal for a day among the patients with tracheostomy. Reintubation within 3 days after extubation was considered unsuccessful.

The primary outcome was post-intubation 90-day survival rate. The secondary outcomes were intubation period, ICU stay, and ventilator withdrawal within 90 days.

### Additional approach

#### Subgroup analysis

We divided the barotrauma group patients into three subgroups: (1) pneumomediastinum/subcutaneous emphysema only, (2) pneumothorax only, and (3) both of pneumomediastinum/subcutaneous emphysema and pneumothorax. The outcome was the 90-day survival rate after barotrauma onset.

#### Landmark analysis

To eliminate immortal bias, we performed a landmark analysis. The landmarks were set at days 7, 14, 21, and 28 after intubation. Barotrauma occurring within 7 days on and before the landmark was defined as the exposure. In each landmark, patients who were extubated before the exposure period and died until the end of that term were excluded. Those who experienced barotrauma occurring within 7 days on and before the landmark were classified as the barotrauma group, and the others were classified as the non-barotrauma group even if they experienced barotrauma after the exposure period. The 90-day plus landmark survival period was compared between the barotrauma and non-barotrauma groups in each landmark analysis. The characteristics of the early (within 7 days) and late (over 7 days) phases after intubation were summarized.

#### Case series

We focused on patients who underwent surgical intervention for COVID-19-related pneumothorax to assess its etiology. Pneumomediastinum/subcutaneous emphysema did not require interventions. As for patients with moderate to large pneumothorax, chest tube insertion was performed. Although air leakage was persistent in patients with pneumothorax, patients with poor physical status were excluded from surgical indication because their lungs were irreversibly damaged and surgical treatment for pneumothorax would not improve their prognosis. In our institution, pulmonologists, ICU Unit and thoracic surgeons discussed the treatment for severe pneumothorax including surgical treatment in each patient and decide the surgical indication.

#### Statistical analysis

Differences between groups with respect to normally and non-normally distributed continuous variables were assessed using the t-test and Mann-Whitney U test as appropriate. Variable changes in the same patient were analyzed using paired t-test. Categorical variables were analyzed using the chi-square test or Fisher’s exact test, as appropriate. The Kaplan–Meier method and log-rank tests were used to analyze the survival curves. Cox proportional hazard analysis was performed by selecting mortality-risk factors reported in previous studies. We performed available case analysis for some factors with missing data (BMI, variables of gas exchange) in the univariate analysis. Complete case analysis was performed in the Cox proportional hazard analysis. All p values were two-sided, and p < 0.05 denoted statistical significance. All statistical analyses were performed using the JMP software (version 16; SAS Institute Inc., Cary, NC, USA).

## Results

### Patient characteristics

Between March 2020 and May 2021, 792 patients with COVID-19 were identified from the database, and 600 patients were excluded because they were treated without mechanical ventilation. Finally, 192 patients were included in this study and 44 (22.9%) experienced barotrauma. One patient received VA-ECMO treatment and was assigned to the non-barotrauma group.

There were no significant differences between the two groups in terms of their characteristics and medical comorbidities (Table 1 and S1 Dataset). Post-intubation gas-exchange variables in the next morning, such as P/F ratio (171.0 vs. 195.7, p = 0.021), pH (7.35 vs. 7.38, p = 0.011), and PaCO_2_ (46.0 mmHg vs. 42.5 mmHg, p = 0.006), exhibited significantly detrimental results with regard to respiratory condition in the barotrauma group (Table 1). On the date of barotrauma onset, the necessary airway pressure, such as peak inspiratory pressure and positive-end expiratory pressure, significantly decreased, and acidosis tended to resolve from the next morning after intubation (Table 2 and S1 Dataset).

**Table 1:** Patient characteristics.

	Barotrauma group (n = 44)	Non-barotrauma group (n = 148)	P value
Age	68.5 (95% CI: 65.2–71.8)	65.2 (63.4–67.0)	0.059
Sex (Male)	28 (63.6%)	110 (74.3%)	0.17
Body mass index (kg/m^2^)	25.2 (23.8–26.6) (n = 44)	25.4 (24.6–26.1) (n = 142)	0.81
Smoking history	22 (50.0%)	86 (58.1%)	0.34
Diabetes mellitus	18 (40.9%)	42 (28.4%)	0.12
Hypertension	23 (52.3%)	82 (55.4%)	0.71
Chronic renal failure	3 (6.8%)	12 (8.1%)	1
Chronic obstructive pulmonary disease	4 (9.1%)	24 (16.2%)	0.33
Variables of gas exchange			
Peak inspiratory pressure (cmH_2_O)	29.2 (27.4–30.9) (n = 35)	28.7 (27.8–29.6) (n = 131)	0.63
Positive end expiratory pressure (cmH_2_O)	12.3 (11.5–13.1) (n = 38)	11.8 (11.4–12.2) (n = 139)	0.30
Tidal volume (mL)	402.1 (378.8–425.4) (n = 38)	413.9 (401.7–426.1) (n = 139)	0.38
P/F ratio	171.0 (152.5–189.5) (n = 38)	195.7 (186.0–205.4) (n = 139)	0.021
pH	7.35 (7.33–7.37) (n = 38)	7.38 (7.37–7.39) (n = 139)	0.011
PaCO_2_ (mmHg)	46.0 (43.8–48.2) (n = 38)	42.5 (41.3–43.6) (n = 139)	0.006

Abbreviation: 95% CI, 95% confidence interval; P/F ratio, the ratio of partial pressure of arterial oxygen to fraction of inspired oxygen; PaCO_2_, partial pressure of carbon dioxide.

**Table 2:** Change in gas-exchange variables.

	Day after intubation	Date of barotrauma	P value
Peak inspiratory pressure (cmH_2_O) (n = 31)	29.4	26.2	0.016
Positive-end expiratory pressure (cmH_2_O) (n = 37)	12.2	10.5	< 0.001
Tidal volume (mL) (n = 33)	402.4	424.1	0.018
P/F ratio (n = 35)	168.7	163.7	0.68
pH (n = 35)	7.35	7.40	< 0.001
PaCO_2_ (mmHg) (n = 35)	45.7	48.4	0.091

Abbreviation: P/F ratio, the ratio of partial pressure of arterial oxygen to fraction of inspired oxygen; PaCO_2_, partial pressure of carbon dioxide.

### Main result

**Fig 1:**
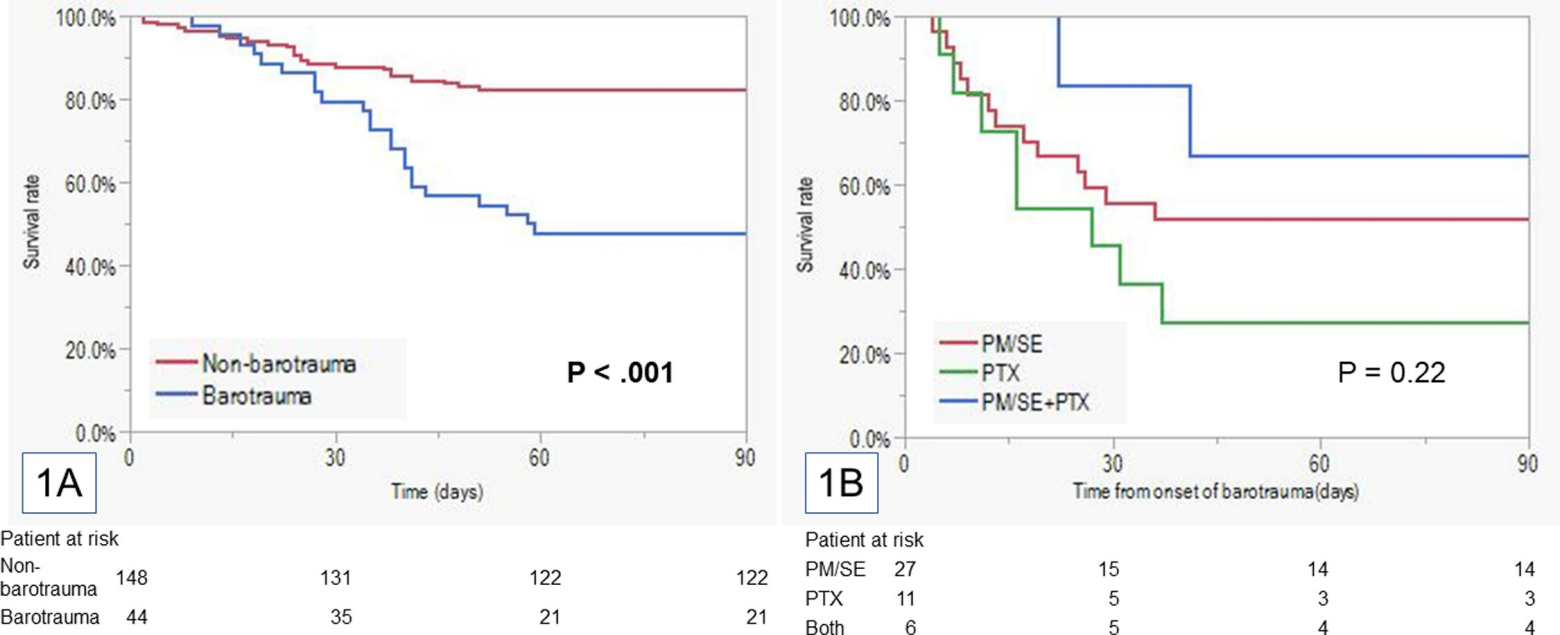
The Kaplan–Meier plot of the survival periods. (**A**) Survival from intubation. (**B**) Survival from barotrauma onset. Abbreviations: PM/SE; pneumomediastinum/subcutaneous emphysema, PTX; pneumothorax, Both; pneumomediastinum/subcutaneous emphysema + pneumothorax.

The 90-day survival rate in the barotrauma group was significantly worse than in the non-barotrauma group (47.7% vs. 82.4%, p < 0.001) (Fig 1A and S1 Dataset). The intubation period (29.5 days [95% confidence interval (CI): 24.3–34.8] vs. 14.5 days [95% CI: 11.7–17.4], p < 0.001) and ICU stay (25.4 days [95% CI: 21.7–29.1] vs. 13.7 days [95% CI: 11.7–15.8], p < 0.001) were longer and fewer patients succeeded in ventilator withdrawal (45.5% vs. 83.8%, p < 0.001) in the barotrauma group (Table 3 and S2 Dataset).

**Table 3:** Treatment and outcome.

	Barotrauma group (n = 44)	Non-barotrauma group (n = 148)	P value
Treatment			
Dexamethasone	42 (95.5%)	134 (90.6%)	0.30
Dexamethasone over standard dose	31 (70.5%)	69 (46.6%)	0.006
Remdesivir	17 (38.6%)	61 (41.2%)	0.76
Tocilizumab	33 (75.0%)	87 (58.8%)	0.051
Prone position	26 (59.1%)	36 (24.3%)	< 0.001
Muscular relaxant	33 (75.0%)	70 (47.3%)	0.001
Outcome			
Intubation period (days)	28.8 (23.6–34.0)	14.3 (11.4–17.1)	< 0.001
ICU stay period (days)	25.4 (21.7–29.1)	13.7 (11.7–15.8)	< 0.001
Ventilator withdrawal	20 (45.5%)	124 (83.8%)	< 0.001
Death	23 (52.3%)	26 (17.6%)	< 0.001
Cause of death			
• COVID-19	19 (82.6%)	20 (76.9%)	0.73
• ARDS, VAP	2 (8.7%)	2 (7.7%)	1
• Others	2 (8.7%; pancreatitis, hemophagocytic syndrome)	4 (15.4%; angina pectoris, sepsis, aspergillosis, lung cancer)	0.67

Abbreviations: 95%CI, 95% confidence interval; ARDS, acute respiratory distress syndrome; COVID-19, coronavirus disease 2019; ICU, intensive care unit; VAP, ventilator-associated pneumonia.

More intensive treatments were administered to the barotrauma group (prescription of dexamethasone > 6 mg/day × 10 days [70.5% vs. 46.6%, p = 0.006], prone positioning [59.1% vs. 24.3%, p < 0.001], and muscular relaxant [75.0% vs. 47.3%, p = 0.001]). The 90-day mortality rate was higher in the barotrauma group (52.3% vs. 17.6%, p < 0.001). In this study, 21 patients survived after barotrauma. At the time of discharge, only 2 patients (9.5%) went back home without oxygen administration, but the other 19 patients (90.5%) were transferred to other hospitals for rehabilitation. Eleven out of 19 patients needed oxygen therapy and 8 patients had tracheostomy tubes (shown in S3 Dataset).

The results of Cox proportional hazard analysis demonstrated that the factors associated with 90-day mortality were barotrauma (hazard ratio [HR]: 2.968 [1.619–5.439], p < 0.001), chronic renal failure (HR: 2.562 [1.098–5.980], p = 0.030), dexamethasone over standard dose (HR: 2.312 [1.200–4.454], p = 0.012), and body mass index (HR: 1.085 [1.017–1.152], p = 0.010) (Table 4 and S1 Dataset).

**Table 4:** The Cox proportional hazard analysis.

	HR	95% CI	P value
Age	1.017	0.987–1.049	0.28
Body mass index	1.085	1.017–1.152	0.010
Smoking history	1.383	0.757–2.526	0.29
Diabetes mellitus	1.453	0.792–2.665	0.23
Chronic renal failure	2.562	1.098–5.980	0.030
Dexamethasone over standard dose	2.312	1.200–4.454	0.012
Barotrauma	2.968	1.619–5.439	< 0.001

Abbreviations: 95% CI, 95% confidence interval; HR, hazard ratio.

### Results of additional analysis

#### Subgroup analysis

There were 27 patients in the pneumomediastinum/subcutaneous emphysema group, 16 in the pneumothorax subgroup, and 6 in the both of pneumomediastinum/subcutaneous emphysema and pneumothorax subgroup, respectively. The 90-day survival rate from the onset of each barotrauma were 51.9%, 27.3%, and 66.7% in the pneumomediastinum/subcutaneous emphysema, pneumothorax, and pneumomediastinum/subcutaneous emphysema + pneumothorax group, respectively (p = 0.22) (Fig 1B and S3 Dataset).

#### Landmark analysis

**Fig 2:**
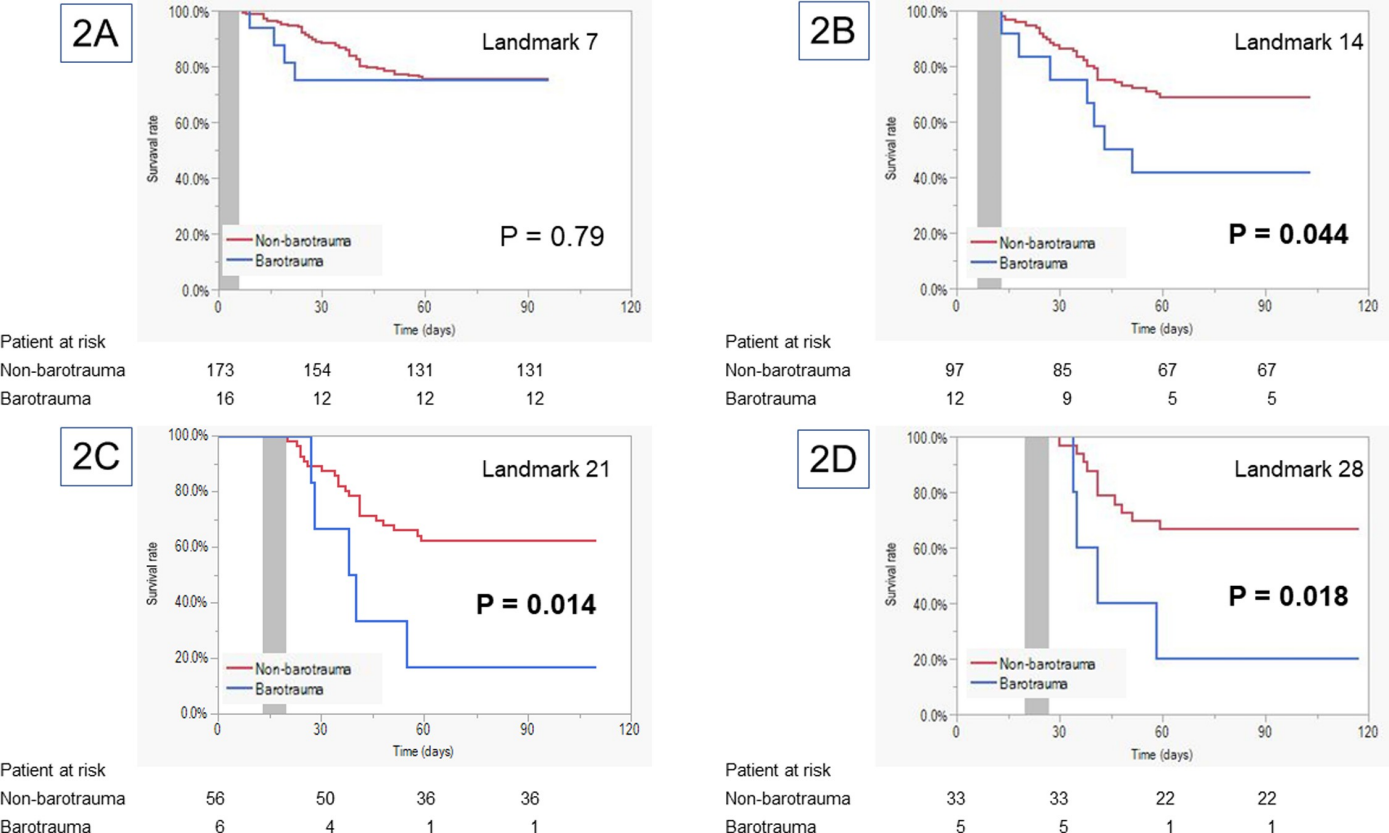
Landmark analysis of the survival periods. The grayish area represents the barotrauma-exposure period. Extubated patients were excluded in each landmark analysis. A: Day 7, B: Day 14, C: Day 21, D: Day 28.

Fig 2 and S4 Dataset, shows the results of landmark survival analysis. In the 7-day landmark analysis, there was no significant difference in the 90-day survival rates from the landmark between the barotrauma and the non-barotrauma groups (n: 16 vs. 173, 75.0% vs. 75.7%, p = 0.79). Nevertheless, the survival rates in the barotrauma group were significantly worse than those in the non-barotrauma group in the 14-, 21-, and 28-day landmark analyses (day 14: n = 12 vs. 97, 41.7% vs. 69.1%, p = 0.044; day 21: n = 6 vs. 56, 16.7% vs. 62.5%, p = 0.014; day 28: n = 5 vs. 33, 20.0% vs. 66.7%, p = 0.018).

There were no significant differences in the details of barotrauma occurrence between the early and late phases. However, the mortality rate was lower in the early phase than in the late phase (25.0% vs. 67.9%, p = 0.011). Among patients with pneumothorax, the complications related to pneumothorax and clinical outcomes were not significantly different (Table 5 and S5 Dataset).

**Table 5:** Comparison of clinical characteristics and outcomes between the early and late phase.

	Early phase (n = 16)	Late phase (n = 28)	P value
PM/SE	11 (68.8%)	16 (57.1%)	0.53
PTX	2 (12.5%)	9 (32.1%)	0.28
Both	3 (18.8%)	3 (10.7%)	0.65
Death	4 (25.0%)	19 (67.9%)	0.01
Cause of death			
• COVID-19	3 (75.0%)	16 (84.2%)	1
• ARDS, VAP	1 (25.0%)	1 (5.3%)	0.32
• others	0 (0%)	2 (10.5%; pancreatitis, hemophagocytic syndrome)	1
In pneumothorax patients (PTX + Both group)	n = 5	n = 12	
Tension pneumothorax	1 (20.0%)	3 (25.0%)	1
Chest tube insertion	2 (40.0%)	9 (75.0%)	0.28
Empyema	0 (0%)	3 (25.0%)	1
Chest tube removal	1 (50.0%)	6 (66.7%)	1
Drainage period (days)	14.5 (95% CI; 3.8–25.2)	9.1 (4.1–14.2)	0.33

Early phase: Within 7 days from intubation; late phase: Over 7 days after intubation. Abbreviation: PM/SE, pneumomediastinum/subcutaneous emphysema; PTX, pneumothorax; Both, pneumomediastinum/subcutaneous emphysema + pneumothorax; COVID-19, coronavirus disease 2019; ARDS, acute respiratory distress syndrome; VAP, ventilator associated pneumonia; 95% CI, 95% confidence interval.

#### Case series

None of the patients in the barotrauma group underwent surgical intervention for barotrauma because their physical status was too poor or the pneumothorax was insignificant. We performed surgical treatment on 3 patients with post-extubation pneumothorax (Table 6). All patients were discharged after surgery with an uneventful clinical course.

**Table 6:** Summary of surgical cases in our institution.

	Case 1 (Male, 69 years old)	Case 2 (Male, 65 years old)	Case 3 (Female, 74 years old)
Body mass index (kg/m^2^)	28.9	26.2	23.2
Past medical history	DM	DM, HT	DM, HT
COVID-19 vaccination	no	no	no
COVID-19 treatment	dexamethasone, mPSL, tocilizumab	PSL pulse	dexamethasone, remdesivir, baricitinib
Preoperative thorombosis (antithrombotic medicine)	PE/DVT (apixaban)	no	DVT (enoxaparin)
Respiratory condition before pneumothorax	room air	room air	HFNC
COVID-19-related pulmonary cyst	no	multiple	single
Combined infection	no	pleural effusion (Candida dubliniensis)	pneumonia, pleural effusion (Stenotrophomonas maltophilia)
Time from COVID-19 onset to pneumothorax (days)	28	30	30
Intraoperative finding	Air leakage and bleeding from the tip of LLL	Air leakage from the bronchial-pleural fistula with necrosis of RUL	Air leakage from the bleb of RML
Surgical approach	VATS wedge resection	VATS coverage of fat pad on the fistula	VATS wedge resection
Postoperative course	Uneventful	uneventful	Several days of HFNC after the surgery
Pathological finding of the leak point	subpleural hematoma	necrotic tissue	bleb and bronchial fistula

Abbreviations: COVID-19, coronavirus disease 2019; DM, diabetes mellitus; HT, hypertention; PSL, predonisolone; PE, pulmonary embolism; DVT, deep venous thrombosis; HFNC, high-flow nasal cannula; LLL, left lower lobe; RUL, right upper lobe; RML, right middle lobe; VATS, video-assisted thoracoscopic surgery.

**Fig 3:**
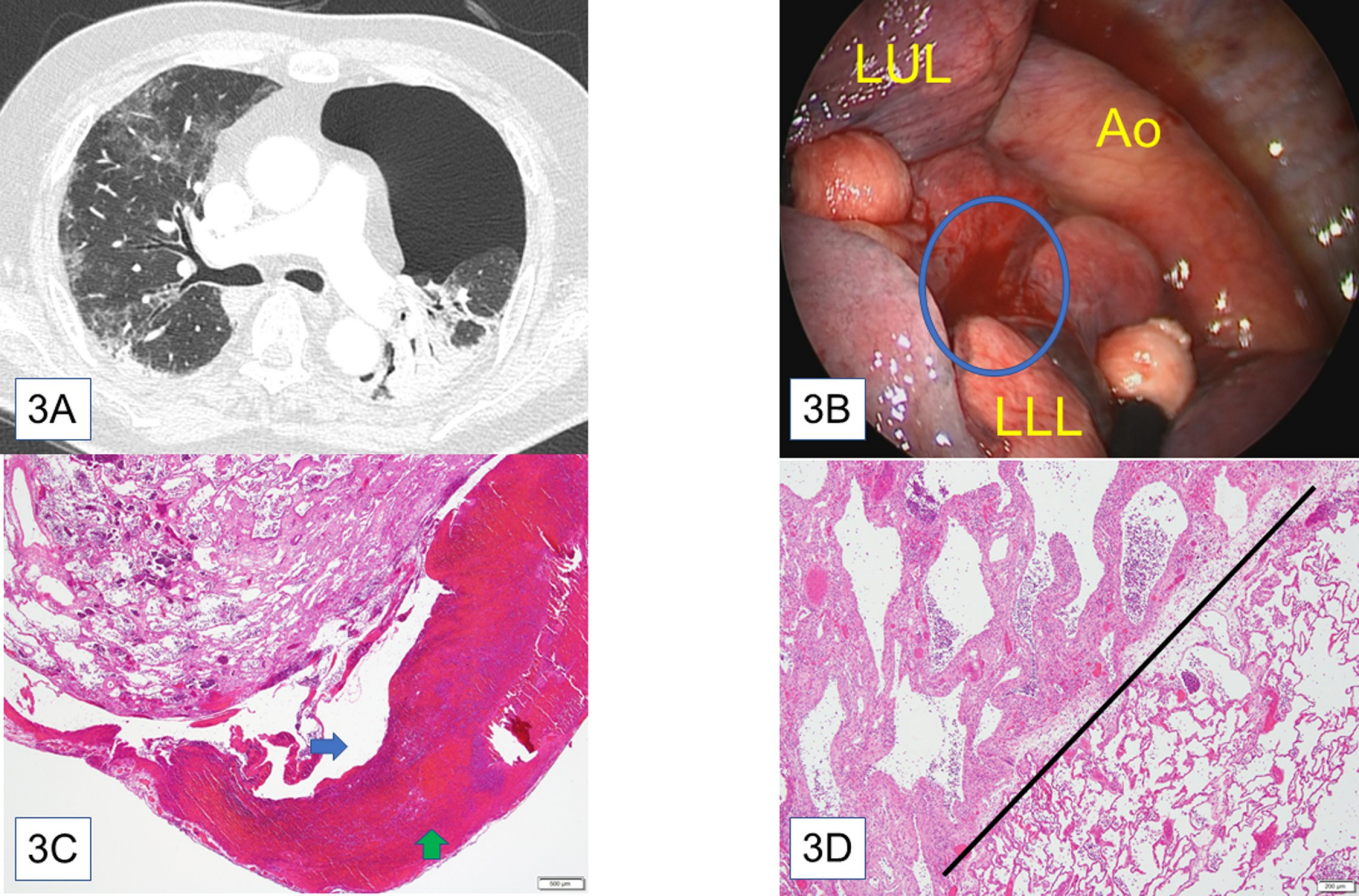
Findings of patient 1. (**A**) Computed tomography at onset of pneumothorax: Ground glass opacities were observed in both lungs. (**B**) Intraoperative findings: Bleeding and air leakage from the tip of the left lower lobe (blue circle). Abbreviations: LUL, left upper lobe; LLL, left lower lobe; Ao, Aorta (**C**) Hematoxylin-eosin staining, ×20: Visceral pleura peeled off by subpleural hematoma (green arrow). The blue arrow indicates air space caused by the dissected visceral pleura. (**D**) Hematoxylin-eosin staining, ×400: Highly inflamed area (left upper side) and intact area (right lower side) are separated by interlobular septa (black line). No histological features associated with pulmonary embolization, such as microvascular thrombi and pulmonary infarction, were observed.

Patient 1: Fibrosis and ground-glass opacities were detected on chest computed tomography (Fig 3A). Thoracoscopy revealed air leakage with bleeding from the tip of the left lower lobe, and partial resection was performed (Fig 3B). Pathological examination revealed hematoma and fibrosis in the subpleural area. The subpleural hematoma tore the visceral pleura at the leak point (Fig 3C). Interstitial edematous granulation tissue, capillary congestion, pneumocyte detachment, intra-alveolar fibrinous exudates, and fibroblastic foci were observed. No hyaline membrane formation was observed. Inflammatory and fibrotic lesions were heterogeneously distributed and divided into a unit of secondary pulmonary lobules (Fig 3D).

**Fig 4:**
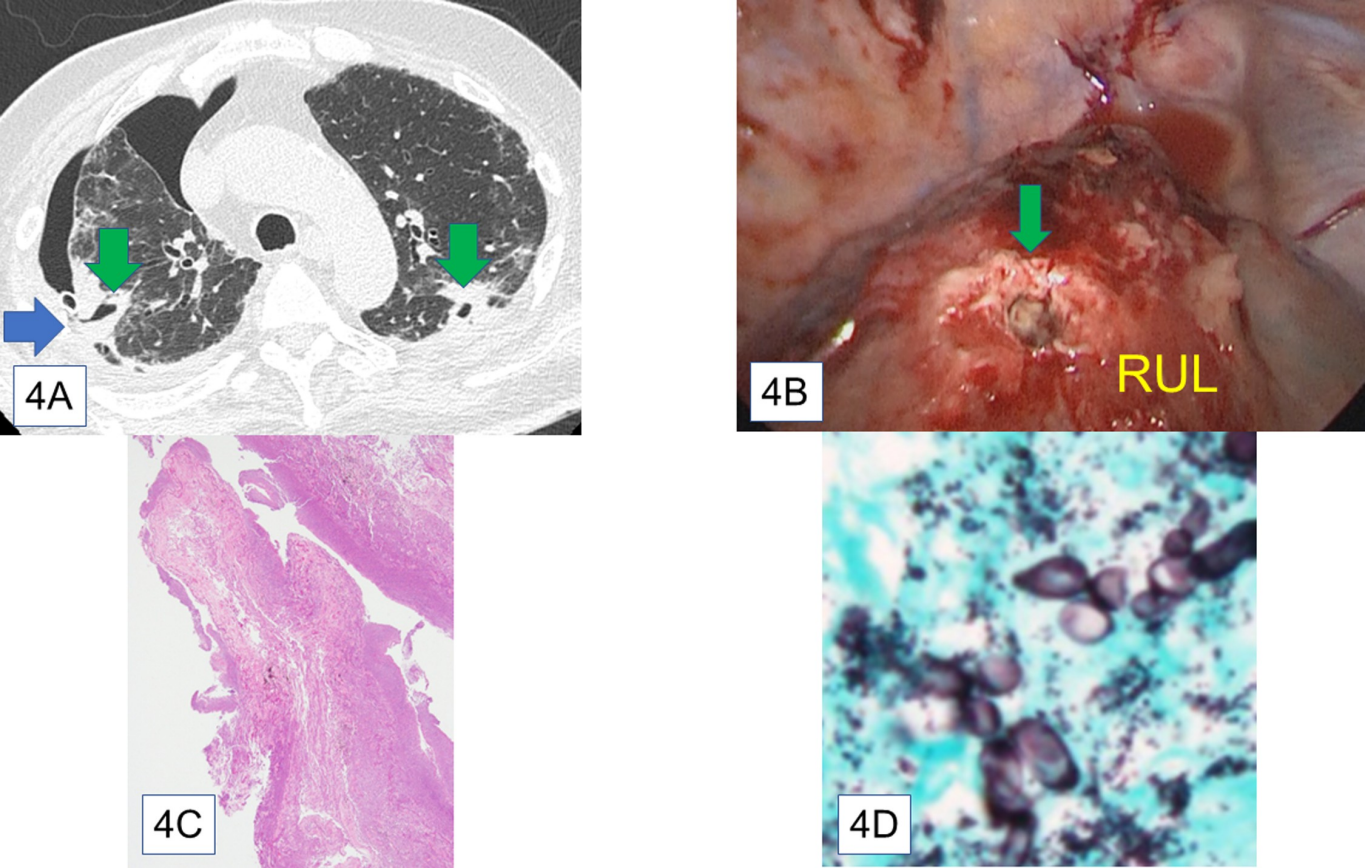
Findings of patient 2. (**A**) Computed tomography at onset of pneumothorax: Multiple cavitary lesions (green arrows) and bronchopleural fistula (blue arrow) in the right upper lobe. (**B**) Intraoperative findings: The right upper lobe exhibited bronchial-pleural fistula (green arrow). Abbreviations: RUL, right upper lobe (**C**) Hematoxylin-eosin staining, ×20: The pulmonary tissue and peripheral bronchus were necrotized with yeast-like fungus growth. (**D**) Grocott’s staining, ×400: Yeast-like fungus was observed.

Patient 2: Chest computed tomography revealed bilateral ground-glass opacities, multiple cavitary lesions, and a bronchopleural fistula in the right upper lobe (Fig 4A). Intraoperatively, the tissues surrounding the fistula and leak point were necrotized (Fig 4B). After curettage of the necrotic tissue, the mediastinal fat pad was sutured to the fistula under thoracoscopy. *Candida dubliniensis* was detected in the cultured pus. Histologically, the resected specimen exhibited fungal growth and necrosis of the peripheral bronchi (Fig 4C and 4D).

**Fig 5:**
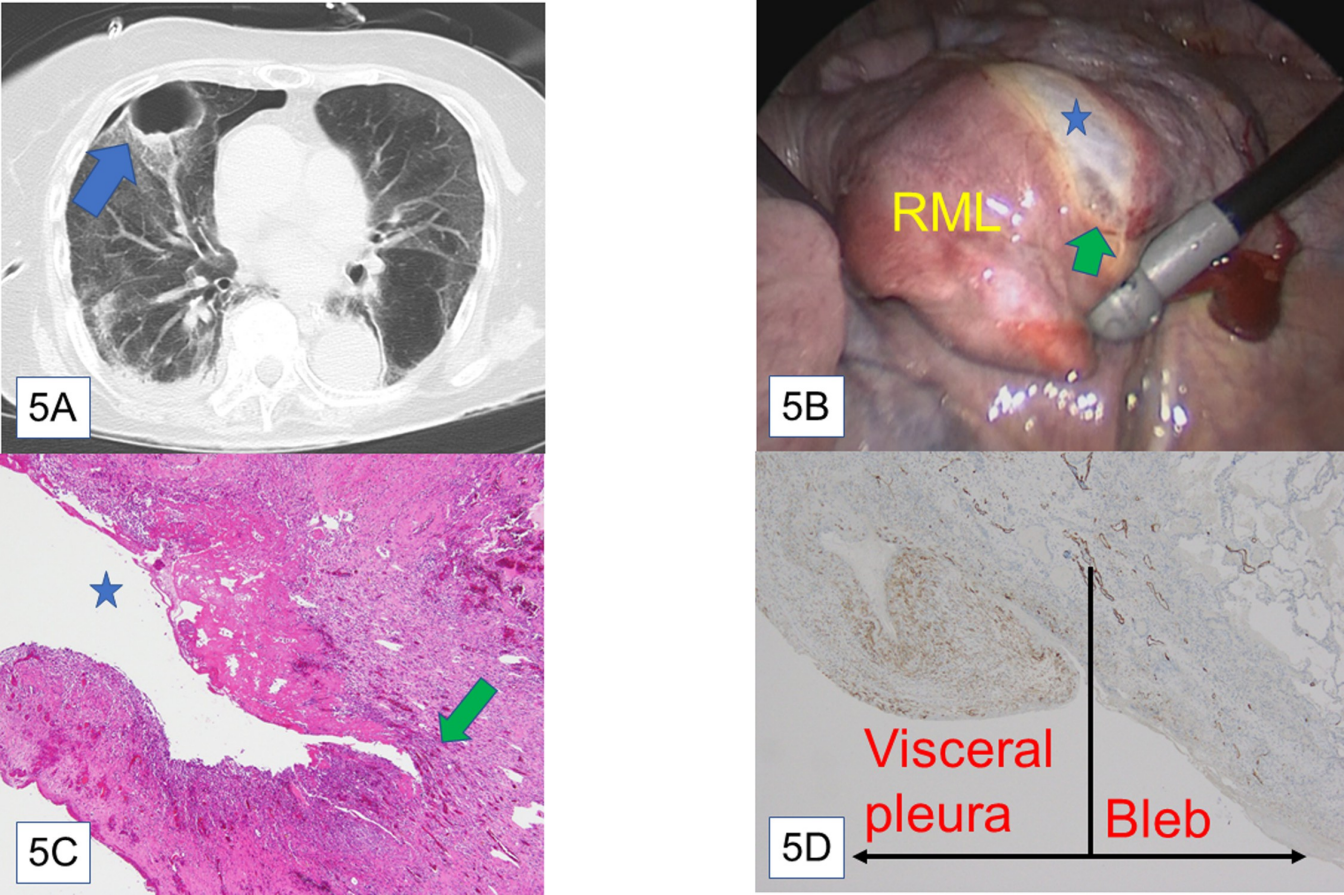
Findings of patient 3. (**A**) Computed tomography at onset of pneumothorax: A giant pulmonary cyst appeared in her right middle lobe (blue arrow). (**B**) Intraoperative findings: Right middle lobe had a bleb (blue star). The green arrow indicates the leak point. Abbreviations: RML, right middle lobe (**C**) Hematoxylin-eosin staining, ×4: The green arrow indicates the bleb-bronchial fistula. The blue star indicates intra-bleb space. (**D**) D2-40 staining, ×4: The black line represents the border between the visceral pleura and inner bleb wall. The mesothelial layer was stained in the visceral pleura but not in the inner bleb wall.

Patient 3: Chest computed tomography at the time of pneumothorax revealed a giant bleb in the right middle lobe that had not been detected before (Fig 5A). After treatment of the hospital-acquired pneumonia and pleuritis caused by *Stenotrophomonas maltophilia*, video-assisted thoracoscopic surgery bullectomy was performed. Air leakage appeared from the fissure of the bleb (Fig 5B). The pathological specimen exhibited a bleb in the inflammatory-thickened visceral pleura communicating with the bronchial fistula (Fig 5C and 5D).

## Discussion

We generated very interesting and informative results regarding COVID-19-related barotraumas. The 90-day survival rate in the barotrauma group was significantly lower than in the non-barotrauma group. Landmark analysis revealed that barotrauma onset over seven days after intubation had a significantly worse impact. On pathological examination, heterogenous lung inflammation and pulmonary cysts were observed although the pathological findings are from patients with post-extubation COVID-19 pneumothorax but not included in the barotrauma group.

It is important to consider the timing of barotrauma because this phenomenon is a kind of side effect of mechanical ventilation and has an inevitable immortal bias. To the best of our knowledge, this is the first study on COVID-19-related barotrauma using a landmark approach, in addition to pathophysiological assessment.

### Perspective of the mechanism

The incidence rate of COVID-19-related barotrauma in this study (22.9%) was as high as previously reported (13.6–26.7%) [4,9,13]. However, the reason we encounter many COVID-19-related barotrauma cases remains unclear [10].

In this study, the results of mechanical ventilation variables indicated that pneumomediastinum/subcutaneous emphysema and pneumothorax in COVID-19 were not caused by “barotrauma” in the narrow sense (pulmonary damage by high airway pressure) [13]. The etiology of pneumomediastinum/subcutaneous emphysema and pneumothorax associated with mechanical ventilation entails volutrauma, biotrauma, atelectrauma, and shear stress, other than “barotrauma” [14–16].

In COVID-19, lung injury is typically characterized by a diffuse alveolar damage pattern; however, other patterns have also been reported [8,17,18]. Histological examination of patient 1 showed interstitial pneumonia that was characterized by a patchy distribution of alveolar edematous thickening and granulation tissue. Such a heterogenous pattern is considered a unique characteristic of COVID-19 lung injuries [19].

Nucci et al. reported that heterogeneous constriction markedly amplifies airway-related shear stress, contributing to ventilator-induced lung injury in a morphological model [20]. This theory is applicable to clinical site [21]. It is estimated that this clearly divided inflammatory lung parenchyma in COVID-19 is a favorable site for shear stress to arise easily because the massive tissue heterogeneity at the alveolar level leads low lung compliance and high pressure during mechanical ventilation. Regions without fibrotic changes receive large regional tidal volumes resulting in high alveolar pressure and barotrauma. It is possible that in these region’s cells release enzymes which together with the enhanced mechanical forces produce local subpleural emphysematous changes which in some cases also lead to pneumothorax [22]. Therefore, we hypothesize that COVID-19-related barotrauma and pulmonary cysts are caused by the highly heterogeneous damage in lung parenchyma some of which potentially reflects progressive regional fibrosis. In patient 1, pulmonary shear stress might have led to bleeding and tearing of the pleura. Moreover, the formation of pulmonary cysts in patient 2 and 3 might potentially had a common etiology. We estimated that air accumulation by fissures in the lung parenchyma due to shear stress leads to pneumatocele and bleb formation, which are known to be a characteristic of COVID-19 pneumonia [23–25]. Bacterial or fungal infection may still trigger the rupture of these pulmonary cysts, which are their comfortable habitats [24].

Although this study demonstrated significant differences in some variables related to mechanical ventilation and arterial blood gas, these differences were too insignificant to be detected clinically. It seems difficult to predict and prevent the occurrence of COVID-19-related barotrauma. Further investigation of the COVID-19-pneumonia model and shear stress is necessary to validate this hypothesis.

### Clinical impact on prognosis

This study demonstrated that those who experienced COVID-19-related barotrauma had higher mortality rates (52.3%) and required more intensive treatment. Barotrauma is a mortality-risk factor. However, the Kaplan–Meier curves in Fig 1B show that most patients with barotrauma did not die immediately after barotrauma onset, and it was not the direct cause of death, as shown in Table 1.

Barotrauma in acute respiratory distress syndrome (ARDS) is a poor prognostic factor and most frequently occurs in the first several days from intubation [2,14]; however, lung structure and function deteriorate according to the duration of ARDS, and barotrauma negatively affects the prognosis in the late stages (> 2 weeks in a previous study) [16]. The time dependency of barotrauma in ARDS is thus implied.

The result of this landmark analysis demonstrated a significantly worse prognosis among those in whom barotrauma occurred over 7 days after intubation; however, early COVID-19-related barotrauma had minimal influence on survival. Fibrosis progression in COVID-19 lung injury has been reported to be accompanied by different disease phases [18]. Hence, we speculate that progressive pulmonary fibrosis in COVID-19 pneumonia leads to worse prognosis, and COVID-19-related barotrauma has a time-dependent nature, as with the usual ARDS.

In COVID-19 cases, fibrosis progression potentially increases the gap between heterogeneity and more shear stress. As mentioned above, shear stress is speculated to play an important role in the incidence of barotrauma in heterogenous inflammatory lung injuries. Therefore, COVID-19-related barotrauma potentially reflects fibrosis progression. In this study, the patients in the barotrauma group received more intensive treatment, such as high-dose steroid prescription, prone positioning and muscular relaxants, than those in the non-barotrauma group. This result indicated that the lung injury caused by COVID-19 pneumonia in the barotrauma group was more severe. We conclude that COVID-19-related barotrauma is a heterogeneous and time-dependent pathology that eventually leads to worse prognosis, especially in the late phase, and an important clinical sign of persistent pulmonary damage.

### Limitations

This study was limited by its single-center, retrospective design and exclusive focus on the Asian population, and the number of pathological specimens obtained was limited.

The results of the main analysis need to be assessed with caution due to the longer intubation period in the barotrauma group. Longer intubation leads to a higher incidence of barotrauma, more intensive treatment, and higher mortality rate. However, using landmark analysis, we compared patients with the same intubation period before the exposure and evaluated the effect of barotrauma during fixed periods. In landmark analysis, the landmark choice may be arbitrary. Most studies on COVID-19 survival or COVID-19-related barotrauma have reported unit results of a week or a month [5]. Actually, COVID-19 patients deteriorated every week. Therefore, a week is considered reasonable for analyzing the prognosis.

Moreover, some mechanical ventilation parameters at the time of intubation (P/F ratio, pH, PaCO_2_) and treatment modalities (prone position and high dose steroids) were different between the barotrauma and the non-barotrauma group, and the data of the barotrauma group tended to be worse than that of the non-barotrauma group as shown in Tables 1 and 2. This is not an interventional study but an observational retrospective study to deepen the understanding of COVID-19-related barotrauma’s characteristics, tendencies, and clinical impacts. Therefore, adjustment of these factors as confounding biases conversely complicated the analyses’ results and interpretation. If an interventional study using barotrauma as the intervention is performed, these mechanical ventilation parameters and treatment modalities should be considered as confounding biases because potential barotrauma group’ patients are possibly sicker.

The Cox proportional hazard analysis in this study might have exhibited a certain level of bias because of the author’s choice and the effect of intermediate variables between barotrauma and medical commodities.

Missing data were processed by available case analysis in the univariate analysis; however, the ratio of missing values is about 10%, therefore we assume the influence would not be significant.

## Conclusions

Barotrauma is a poor prognostic factor for ventilated patients with COVID-19; however, COVID-19-related barotrauma has a potential time-dependent impact. In particular, over seven days after intubation, the incidence of barotrauma was a risk factor. Heterogenous lung inflammation may be a key finding in the mechanism of barotrauma, and barotrauma may be an important clinical sign of COVID-19-induced lung destruction.

## Supporting information

S1 Dataset The clinical characteristics of all intubated COVID-19 patients. (XLSX)

S2 Dataset The treatment modalities and outcomes. (XLSX)

S3 Dataset The clinical outcomes among the pneumomediastinum/subcutaneous emphysema group, the pneumothorax group, and the pneumomediastinum/subcutaneous emphysema + pneumothorax group. (XLSX)

S4 Dataset The clinical outcomes of patients classified by the landmark approach. The tab 2A, 2B, 2C, and 2D correspond to the 7-, 14-, 21-, and 28-day landmark analysis, respectively.
(XLSX)

S5 Dataset The clinical and outcomes between the early and late phase. (XLSX)
